# Aminoacylation and translational quality control strategy employed by leucyl-tRNA synthetase from a human pathogen with genetic code ambiguity

**DOI:** 10.1093/nar/gkt741

**Published:** 2013-08-22

**Authors:** Xiao-Long Zhou, Zhi-Peng Fang, Zhi-Rong Ruan, Meng Wang, Ru-Juan Liu, Min Tan, Fabrizio Maria Anella, En-Duo Wang

**Affiliations:** Center for RNA Research, State Key Laboratory of Molecular Biology, Institute of Biochemistry and Cell Biology, Shanghai Institutes for Biological Sciences, The Chinese Academy of Sciences, 320 Yue Yang Road, Shanghai 200031, China

## Abstract

Aminoacyl-tRNA synthetases should ensure high accuracy in tRNA aminoacylation. However, the absence of significant structural differences between amino acids always poses a direct challenge for some aminoacyl-tRNA synthetases, such as leucyl-tRNA synthetase (LeuRS), which require editing function to remove mis-activated amino acids. In the cytoplasm of the human pathogen *Candida albicans*, the CUG codon is translated as both Ser and Leu by a uniquely evolved *Ca*tRNA^Ser^(CAG). Its cytoplasmic LeuRS (*Ca*LeuRS) is a crucial component for CUG codon ambiguity and harbors only one CUG codon at position 919. Comparison of the activity of *Ca*LeuRS-Ser^919^ and *Ca*LeuRS-Leu^919^ revealed yeast LeuRSs have a relaxed tRNA recognition capacity. We also studied the mis-activation and editing of non-cognate amino acids by *Ca*LeuRS. Interestingly, we found that *Ca*LeuRS is naturally deficient in tRNA-dependent pre-transfer editing for non-cognate norvaline while displaying a weak tRNA-dependent pre-transfer editing capacity for non-cognate α-amino butyric acid. We also demonstrated that post-transfer editing of *Ca*LeuRS is not tRNA^Leu^ species-specific. In addition, other eukaryotic but not archaeal or bacterial LeuRSs were found to recognize *Ca*tRNA^Ser^(CAG). Overall, we systematically studied the aminoacylation and editing properties of *Ca*LeuRS and established a characteristic LeuRS model with naturally deficient tRNA-dependent pre-transfer editing, which increases LeuRS types with unique editing patterns.

## INTRODUCTION

Aminoacyl-tRNA synthetases (aaRSs) are essential components required to establish the genetic code during protein biosynthesis by coupling specific amino acids with their cognate tRNAs in a two-step aminoacylation reaction (1,2). This process requires amino acid activation by condensation with ATP to form the aminoacyl-adenylate (aa-AMP) and pyrophosphate; the activated amino acid is then transferred to the cognate tRNA to yield the aminoacyl-tRNA (aa-tRNA), which is then transferred to the protein biosynthesis machinery as a building block (1). Aminoacylation of tRNA requires adequate efficiency and accuracy, which requires tightly regulated control of the speed of the aa-tRNA production for the ribosome and the risk of generation of aberrant aa-tRNA pairs (3–5). Transfer RNA always harbors various identity determinants and/or anti-determinants, facilitating selection of the correct tRNA from a large pool of tRNA species (6). However, the specificity of aaRS is greatly challenged by the presence of various types of amino acids and their analogues and the fact that amino acids differ only in the side-chain. AaRSs that do not show an overall selectivity above 1 in 3000 are predicted to require some form of proofreading (editing) mechanism to maintain sufficient accuracy during aa-tRNA synthesis (5,7,8). Editing activity has evolved in half of the currently identified aaRSs to remove any aberrantly produced aa-AMP (pre-transfer editing) and/or aa-tRNA (post-transfer editing). This is an essential checkpoint that ensures translational fidelity (5). Pre-transfer editing can be further divided into tRNA-independent and tRNA-dependent pre-transfer editing. In tRNA-independent pre-transfer editing, the non-cognate aa-AMP is hydrolyzed into the amino acid and AMP molecules without the presence of cognate tRNA, whereas in tRNA-dependent pre-transfer editing, aa-AMP hydrolysis is triggered by the addition of the cognate tRNA (9–11). Mis-translation due to the impairment or loss of editing activity can lead to ambiguity of the proteome, having a seriously negative effect on the cellular function of most organisms and causing neuron-degeneration in a mouse model (12).

Leucyl-tRNA synthetase (LeuRS) is a large multi-domain class Ia aaRS with both aminoacylation activity to generate Leu-tRNA^Leu^ and editing activity to clear non-cognate aa-AMP and aa-tRNA (13). It can be divided into bacterial and archaeal/eukaryotic types based on primary sequence and domain location (14). Both types of LeuRSs usually consist of a Rossmann-fold domain (for amino acid activation and aminoacylation), an α-helix bundle, a C-terminal domain (for tRNA binding) and a CP1 domain (for editing) (15–17). Extensive studies of various LeuRS species all found that non-cognate norvaline (Nva) is the most significantly mis-activated amino acid among all the non-cognate amino acids tested, including Ile, Val, Met and α-amino butyric acid (ABA). For instance, compared with cognate Leu, Nva is mis-activated by *Aquifex aeolicus* LeuRS (*Aa*LeuRS) (9), *Saccharomyces cerevisiae* LeuRS (*Sc*LeuRS) (18), human cytoplasmic LeuRS (hcLeuRS) (19), *Mycoplasma mobile* LeuRS (*Mm*LeuRS) (20), human mitochondrial LeuRS (hmtLeuRS) (unpublished data) 72-, 105-, 100-, 122- and 180-fold less efficiently, respectively. Nva is a non-proteinogenic amino acid differing from Leu only by the absence of a side-chain methyl group. Nva is naturally present *in vivo* and is a by-product of the Leu biosynthesis pathway (21). Its synthesis is predominantly related to an imbalance in the synthesis of the branched-chain amino acids under pyruvate-high conditions. In addition, Nva significantly accumulates immediately after a shift from aerobic culture conditions to oxygen limitation at high glucose concentrations (22). Therefore, the amount of Nva is dynamic and varies according to the environment. The incorporation of Nva in proteins at Leu codons has been clearly demonstrated. It has been reported to be a natural component of an antifungal peptide of *Bacillus subtilis* (23) and can be intentionally inserted into heterologous proteins by culturing *Escherichia coli* in the presence of Nva (US patent, Nov 7, 1989, 4879223). Accompanied by conditions of an elevated ratio of available Nva to Leu in the medium, increasing mis-incorporation of Nva at Leu codons has been observed in recombinant human hemoglobin produced in *E. coli* as a result of mis-aminoacylation of tRNA^Leu^ by *E. coli* LeuRS (*Ec*LeuRS) (24). It is proposed that Nva replacement may disrupt the correct folding and assembly of hemoglobin and other proteins (24). All this evidence suggests that Nva mis-activation by LeuRS is a non-artificial event that occurs *in vivo*, and that mis-charged Nva-tRNA^Leu^ can be accommodated and used by the ribosome. Therefore, editing of Nva by LeuRS seems to be essential for the correct functioning of organisms.

Based on significant mis-activation of Nva, editing catalyzed by LeuRS (with a functional CP1 domain) has been shown to be one of the most interesting editing mechanisms. This process is predominantly mediated by three diverse pathways (tRNA-independent, tRNA-dependent pre-transfer and post-transfer editing) (10). Both types of LeuRS critically depend on the editing active site embedded in the CP1 domain to perform post-transfer editing (15–17,25). However, *Mm*LeuRS harbors only tRNA-independent pre-transfer editing activity owing to its natural lack of the CP1 domain (20). Another example of a unique LeuRS is hmtLeuRS, which possesses a degenerate editing active site in the CP1 domain as well as defunct post-transfer editing (26) and tRNA-dependent pre-transfer editing activities (unpublished data). Combining site-directed mutagenesis and AMP formation methodology, the contribution of different pathways to the overall editing process can be quantified (9,10,19). Strikingly, there are quantitative and species-specific differences in the contribution of a specific pathway to the total editing activity of a LeuRS (9,10,19). To evaluate the significance of each mechanism, we have attempted to generate LeuRSs lacking one or more editing mechanism(s); to date, two types have been successfully established. One type contains LeuRSs with abolished post-transfer editing activity, obtained by introducing mutations at key residues (e.g. *Ec*LeuRS-T252R, *Aa*LeuRS-T273R, *Aa*LeuRS-D373A, *Sc*LeuRS-D419A, hcLeuRS-D399A, *Giardia lamblia (Gl)* LeuRS-D444A) (9,10,18,19,27) or by the inclusion of a small molecule inhibitor (AN2690) of the CP1 editing domain (10). The second type includes LeuRSs for which both the post-transfer editing and tRNA-dependent pre-transfer editing activities (*Ec*LeuRS-Y330D, *Aa*LeuRS-Y358D) have been abolished (10). Our aim was to determine whether a LeuRS with defective tRNA-dependent pre-transfer editing activity but intact post-transfer editing would produce mis-charged tRNAs. However, extensive efforts to establish such a LeuRS model failed.

The protein biosynthesis machinery of *Candida albicans* is of great interest, not only because it is a human pathogen but also in its cytoplasm, the universal Leu codon CUG is translated as both Ser (97%) and Leu (3%) (28,29). This genetic code alteration is mediated by a uniquely evolved tRNA, which bears a CAG anti-codon [*C. albicans* tRNA^Ser^(CAG), *Ca*tRNA^Ser^] and can be aminoacylated either with Ser by *C. albicans* seryl-tRNA synthetase (*Ca*SerRS) or with Leu by leucyl-tRNA synthetase (*Ca*LeuRS) (29). Therefore, the proteome of *C. albicans* is ambiguous with some proteins exhibiting differences in primary sequences. For example, a key player in CUG reassignment, *Ca*SerRS, has two isoforms (SerRS-Leu^197^ and SerRS-Ser^197^). The residue at position 197 is located at the SerRS dimer interface, and replacement of Ser by Leu at this site induces a local structural rearrangement, leading to a slightly higher (27%) activity of SerRS-Leu^197^ compared with SerRS-Ser^197^ (30). These data indicate that distribution of the CUG codon and its ambiguity is not random and has potential significance. *Ca*LeuRS is another critical molecule in the CUG reassignment in *C. albicans*, which charges *Ca*tRNA^Ser^ with Leu to produce Leu-*Ca*tRNA^Ser^. *Ca*LeuRS comprises 1098 residues and has a molecular mass of 126 kDa. A single CUG codon is present at position of 919 of *Ca*LeuRS, which is located at the C-terminal domain. Thus, *Ca*LeuRS should also have two isoforms, *Ca*LeuRS-Ser^919^ and *Ca*LeuRS-Leu^919^. Based on the decoding rule of *C. albicans* (28,29), *Ca*LeuRS exists mainly as *Ca*LeuRS-Ser^919^ (∼97%), and this was used here as the wild-type form.

In this study, we compared the activity of two LeuRS isoforms and analyzed the cross-species tRNA^Leu^ recognition and editing capacity of *Ca*LeuRS. Interestingly, we showed that *Ca*LeuRS is naturally deficient in tRNA-dependent pre-transfer editing activity but with obvious tRNA-independent pre-transfer editing and efficient post-transfer editing of Nva. However, it harbored a measurable level of tRNA-dependent pre-transfer editing of ABA when specific tRNA was present, although editing of ABA seemed not to be a necessity, as the rejection of ABA was efficient at the aminoacylation active site. Furthermore, post-transfer editing of *Ca*LeuRS was not tRNA^Leu^ species-specific but was functional for mis-charged *Ca*tRNA^Ser^(CAG), being recognized by other eukaryotic LeuRSs.

## MATERIALS AND METHODS

### Materials

l-leucine (Leu), l-norvaline (Nva), l-isoleucine (Ile), l-valine (Val), l-methionine (Met), l-serine (Ser), ABA, dithiothreitol (DTT), ATP, CTP, GTP, UTP. 5′-GMP, tetrasodium pyrophosphate, inorganic pyrophosphate, ATP, Tris–HCl, MgCl_2_, NaCl, yeast total tRNA and activated charcoal were purchased from Sigma (St. Louis, MO, USA). [^3^H]Leu, [^32^P]tetrasodium pyrophosphate and [α-^32^P]ATP were obtained from PerkinElmer Life Sciences (Boston, MA, USA). Pfu DNA polymerase, a DNA fragment rapid purification kit and a plasmid extraction kit were purchased from YPH Company (China). The KOD-plus mutagenesis kit was obtained from TOYOBO (Japan). T4 ligase, nuclease S1 and restriction endonucleases were obtained from MBI Fermentas (Pittsburgh, PA, USA). Phusion high-fidelity DNA polymerase was purchased from New England Biolabs (Ipswich, MA, USA). Ni^2+^-NTA Superflow was purchased from Qiagen, Inc. (Germany). Polyethyleneimine cellulose plates were purchased from Merck (Germany). Pyrophosphatase (PPiase) was obtained from Roche Applied Science (China). The dNTP mixture was obtained from TaKaRa (Japan). Oligonucleotide primers were synthesized by Biosune (China). *Escherichia coli* BL21 (DE3) cells were purchased from Stratagene (USA).

### Gene cloning, mutagenesis and protein expression

The *C. albicans* genome was kindly provided by Prof. Jiang-Ye Chen of our institute and was used as the template for amplifying genes encoding *Ca*LeuRS, *C. albicans* SerRS (*Ca*SerRS) and *C. albicans* mitochondrial LeuRS (*Ca*mtLeuRS). Gene sequences of *Ca*LeuRS, *Ca*SerRS and *Ca*mtLeuRS were obtained from the Candida Genome Database (http://www.candidagenome.org/). *Ca*LeuRS, *Ca*SerRS and *Ca*mtLeuRS genes were cloned into pET28a at the *Nhe*I and *Xho*I sites with N-terminal His_6_-tag (the mitochondrial targeting sequence of *Ca*mtLeuRS had been removed). Plasmids containing *Ec*LeuRS (10), *Sc*LeuRS (18) and *Pyrococcus horikoshii* LeuRS (*Ph*LeuRS) (31) were constructed previously. The *E. coli* tRNA(m^1^G37) methyltransferase (TrmD) gene was amplified from the *E. coli* genome and inserted between the *EcoR*I and *Xho*I of sites of pET28a. The plasmid expressing *E. coli* tRNA nucleotidyltransferase (CCase) was provided by Dr. Gilbert Eriani (Strasbourg, CNRS, France). Mutation at Asp^422^ of the *Ca*LeuRS gene was performed with the KOD-plus mutagenesis kit according to the manufacturer’s instructions. Asp^422^ corresponds to Asp^373^, Asp^419^, Asp^444^ and Asp^399^ of *Aa*LeuRS, *Sc*LeuRS, *Gl*LeuRS and hcLeuRS, respectively, which are crucial for post-transfer editing of these LeuRSs (9,10,18,19,27). The CTG and TCG codons at position 919 in the *Ca*LeuRS gene were used to over-express the gene encoding *Ca*LeuRS-Leu^919^ and *Ca*LeuRS-Ser^919^, respectively. All constructs were confirmed by DNA sequencing. *E. coli* BL21 (DE3) was transformed with various constructs. A single colony of each of the transformants was chosen and cultured in 500 ml of 2 × YT medium at 37°C. When the cells reached mid-log phase (A_600_ = 0.6), expression of the recombinant proteins was induced by the addition of 0.2 mM isopropyl-1-thio-β-D-galactopyranoside for 8 h at 22°C. Protein purification was performed according to a previously described method (32).

### tRNA gene cloning, transcription and methylation

*Ca*tRNA^Leu^(UAA) and *Ca*tRNA^Ser^(CAG) genes were cloned between the *Pst*I and *EcoR*I sites of pTrc99b with an N-terminal T7 promoter. Detailed T7 *in vitro* run-off transcription of *Ca*tRNA^Leu^ and *Ca*tRNA^Ser^ has been described previously (33). The amino acid accepting activities of *Ca*tRNA^Leu^(UAA) or *Ca*tRNA^Ser^(CAG) are 1390 and 1208 pmol/A_260_, respectively. The methyl group of m^1^G37 of *Ca*tRNA^Ser^ is a critical element for recognition by LeuRS (29). The purified *Ca*tRNA^Ser^ transcript was methylated at position G37 with *E. coli* TrmD (34) in a mixture containing 0.1 M Tris–HCl (pH 8.0), 1 mM DTT, 0.1 mM EDTA, 6 mM MgC1_2_, 24 mM NH_4_C1, 7.5 μg of bovine serum albumin, 5 μM *Ca*tRNA^Ser^ transcript, 100 μM S-adenosylmethionine, 1 U/μl RNase inhibitor and 10 μM TrmD at 37°C for 1.5 h. Approximately 45% of transcripts were methylated in this reaction as estimated in a control experiment with ^3^H-labeled S-adenosylmethionine. m^1^G37-*Ca*tRNA^Ser^ was ethanol-precipitated at −20°C after phenol/chloroform extraction (twice) and dissolved in 5 mM MgCl_2_. All *Ca*tRNA^Ser^ used in this study refers to m^1^G37-*Ca*tRNA^Ser^. Transcribed or over-expressed *E. coli* tRNA^Leu^(GAG) (*Ec*tRNA^Leu^) and human cytoplasmic tRNA^Leu^(CAG) (hctRNA^Leu^) were obtained according to methods described elsewhere, and their amino acid accepting activity was ∼1500 pmol/A_260_ (19,35).

### ^32^P-labeling of *Ca*tRNA^Leu^ or *Ca*tRNA^Ser^

^32^P-labeling of *Ca*tRNA^Leu^ or *Ca*tRNA^Ser^ was performed at 37°C in a mixture containing 60 mM Tris–HCl (pH 8.0), 12 mM MgCl_2_, 15 μM *Ca*tRNA^Leu^ or *Ca*tRNA^Ser^, 0.5 mM DTT, 15 μM ATP, 50 μM tetrasodium pyrophosphate, 0.666 μM [α-^32^P]ATP and 10 μM CCase for 5 min. Finally, 0.8 U/μl PPiase was added to the mixture for 2 min. Phenol/chloroform extraction of [^32^P]*Ca*tRNA^Leu^ and [^32^P]*Ca*tRNA^Ser^ was conducted twice, and the product was dissolved in 5 mM MgCl_2_.

### *In vitro* activity assays

ATP-PPi exchange measurement was carried out at 30°C in a reaction mixture containing 60 mM Tris–HCl (pH 7.5), 10 mM MgCl_2_, 2 mM DTT, 4 mM ATP, 2 mM [^32^P]tetrasodium pyrophosphate, 1 mM Leu or 50 mM non-cognate ABA, Nva, Val, Ile, Met, Ser and 20 nM *Ca*LeuRS. The kinetics of amino acid activation were measured in the presence of Leu (3–1000 μM) or Nva (0.3–50 mM) or ABA (3–940 mM). Samples of the reaction mixture were removed at specific time-points, added to 200 μl of quenching solution containing 2% activated charcoal, 3.5% HClO_4_ and 50 mM tetrasodium pyrophosphate and mixed by vortexing for 20 s. The solution was filtered through a Whatman GF/C filter, followed by washing with 20 ml of 10 mM tetrasodium pyrophosphate solution and 10 ml of 100% ethanol. The filters were dried, and [^32^P]ATP was counted using a scintillation counter (Beckman Coulter).

Aminoacylation of *Cat*RNA^Leu^ with Leu was performed in a reaction mixture containing 60 mM Tris–HCl (pH 7.5), 10 mM MgCl_2_, 2 mM DTT, 4 mM ATP, 10 μM *Ca*tRNA^Leu^, 20 μM [^3^H]Leu and 20 nM *Ca*LeuRS at 30°C. The kinetics of *Ca*LeuRS aminoacylation were measured in the presence of *Ca*tRNA^Leu^ (0.6–15.8 μM) or transcribed or over-expressed *Ec*tRNA^Leu^ (0.6–10 μM) or yeast total tRNA (0.2–6 μM) or over-expressed hctRNA^Leu^ (0.2–6 μM) or transcribed hctRNA^Leu^ (0.6–10 μM).

Mis-aminoacylation of [^32^P]*Ca*tRNA^Leu^ with Nva or ABA was carried out at 30°C in a reaction mixture containing 60 mM Tris–HCl (pH 7.5), 10 mM MgCl_2_, 2 mM DTT, 4 mM ATP, 5 μM ‘cold’ *Ca*tRNA^Leu^, 1 μM [^32^P]*Ca*tRNA^Leu^, 20 mM Nva or 376 mM ABA and 1 μM *Ca*LeuRS or *Ca*LeuRS-D422A. Samples at specific time-points were taken for ethanol precipitation with NaAc (pH 5.2) at −20°C overnight. The precipitated samples were centrifuged (10 000*g*) at 4°C for 30 min, dried at room temperature for 30 min and digested with 6 μl of nuclease S1 (25 U) for 2 h at 37°C. After treatment with nuclease S1, aminoacyl-[^32^P]tRNA should produce aminoacyl-[^32^P]AMP and free [^32^P]tRNA should produce [^32^P]AMP. Samples (2 μl) of the digestion mixture were loaded and separated by thin layer chromatography (TLC) in 0.1 M NH_4_Ac and 5% acetic acid. Known amounts of [α-^32^P]ATP were diluted and loaded onto the TLC plate for the purposes of quantification. The plates were visualized by phosphorimaging, and the data were analyzed using Multi-Gauge Version 3.0 software (FUJIFILM). Measurement of Nva-[^32^P]*Ca*tRNA^Ser^ synthesis by *Ca*LeuRS and *Ca*LeuRS-D422A was performed using a similar procedure, except [^32^P]*Ca*tRNA^Ser^ was used as a substrate. Mis-aminoacylation of [^32^P]*Ca*tRNA^Ser^ with Leu by various LeuRSs was carried out in a reaction mixture containing 60 mM Tris–HCl (pH 7.5), 10 mM MgCl_2_, 2 mM DTT, 4 mM ATP, 5 μM ‘cold’ *Ca*tRNA^Ser^, 1 μM [^32^P]*Ca*tRNA^Ser^, 40 mM Leu and 1 μM *Ca*LeuRS, *Ca*LeuRS-D422A, *Ca*mtLeuRS, *Ec*LeuRS, *Sc*LeuRS, hcLeuRS and *Ph*LeuRS at 37°C.

Preparation of Nva-[^32^P]*Ca*tRNA^Leu^ or Nva-[^32^P]*Ca*tRNA^Ser^ was carried out with editing-deficient *Sc*LeuRS-D419A or *Ca*LeuRS-D422A, respectively, in a reaction mixture, which was identical to that used for mis-aminoacylation. Post-transfer editing of pre-formed Nva-[^32^P]*Ca*tRNA^Leu^ was performed in a reaction mixture containing 60 mM Tris–HCl (pH 7.5), 10 mM MgCl_2_, 1 μM Nva-[^32^P]*Ca*tRNA^Leu^ and 30 nM *Ca*LeuRS or *Ca*LeuRS-D422A at 30°C. Post-transfer editing of pre-formed Nva-[^32^P]*Ca*tRNA^Ser^ was performed in a reaction containing 60 mM Tris–HCl (pH 7.5), 10 mM MgCl_2_, 1 μM Nva-[^32^P]*Ca*tRNA^Ser^ and 100 nM *Ca*LeuRS or *Ca*LeuRS-D422A at 30°C. After nuclease S1digestion, the amount of hydrolyzed mis-charged [^32^P]tRNAs was assayed by TLC according to the procedure described for mis-aminoacylation.

The AMP formation assay was carried out at 30°C in a reaction mixture containing 60 mM Tris–HCl (pH 7.5), 10 mM MgCl_2_, 5 mM DTT, 10 U/ml PPiase, 15 mM Nva or 350 mM ABA, 3 mM [α-^32^P]ATP and 0.2 μM *Ca*LeuRS in the presence or absence of 15 μM transcribed *Ca*tRNA^Leu^, or 5 μM transcribed or over-expressed *Ec*tRNA^Leu^ or hctRNA^Leu^. Samples (1.5 µl) were quenched in 6 µl of 200 mM NaAc (pH 5.0). The quenched aliquots (1.5 µl of each sample) were spotted onto polyethyleneimine cellulose plates pre-washed with water. Separation of Nva/ABA-[α-^32^P]AMP, [α-^32^P]AMP and [α-^32^P]ATP was performed in 0.1 M NH_4_Ac and 5% acetic acid. Quantification of [α-^32^P]AMP was achieved by densitometry in comparison with [α-^32^P]ATP samples of known concentrations.

## RESULTS

### *Ca*LeuRS-Leu^919^ is more active than *Ca*LeuRS-Ser^919^

**Figure 1.**
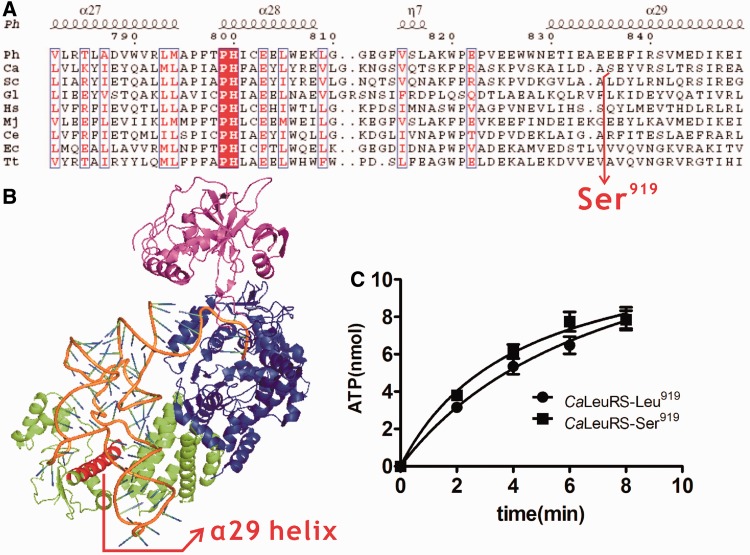
Location of residue 919 and its effect on amino acid activation in *Ca*LeuRS. (**A**) Primary sequence alignment of LeuRSs from three domains of life with position of 919 indicated. Amino acid sequences homologous to those from α27 to α29 helix of *Ph*LeuRS are aligned. (**B**) Crystal structure of *Ph*LeuRS-tRNA^Leu^ structure showing the position of 919-containing α29 helix. (**C**) Amino acid activation measurement of *Ca*LeuRS-Leu^919^ (black circle) and *Ca*LeuRS-Ser^919^ (black square). Ph, *Pyrococcus horikoshii*; Ca, *C. albicans*; Sc, *S. cerevisiae*; Gl, *Giardia lamblia*; Hs, *Homo sapiens*; Mj, *Methanococcus jannaschii*; Ce, *Caenorhabditis elegans*; Ec, *E. coli*; Tt, *Thermus thermophiles*.

Determination of the crystal structure of the *Ph*LeuRS-tRNA^Leu^ complex (Protein Data Bank, PDB 1WZ2) shows that the amino acid at position 919 of archaeal/eukaryotic LeuRSs is located in the α29 helix of the C-terminal domain (Figure 1A and B). The primary sequence of the 919-containing α29 helix is not conserved; thus, it is difficult to identify its homologous site in the crystal structure of *Ph*LeuRS. The CUG codon in *E. coli* is uniformly translated as Leu. Therefore, we introduced CTG and TCG codons at this position in the *Ca*LeuRS gene to facilitate expression of *Ca*LeuRS-Leu^919^ and *Ca*LeuRS-Ser^919^, respectively, in *E. coli*.

No differences were observed in amino acid activation by *Ca*LeuRS-Ser^919^ and *Ca*LeuRS-Leu^919^ (Figure 1C), indicating that Leu or Ser insertion at this position has no direct effect on the structure or function of the aminoacylation active site located in the Rossmann-fold domain. This is consistent with the fact that residue 919 is spatially distant from the aminoacylation active site (>50 Å in the *Ph*LeuRS-tRNA^Leu^ structure) (Figure 1B). Subsequent comparisons of the aminoacylation kinetics of *Ca*LeuRS-Ser^919^ and *Ca*LeuRS-Leu^919^ revealed that *Ca*LeuRS-Leu^919^ displayed a higher *K*_m_ (2.91 ± 0.37 μM) and a higher *k_cat_* (0.62 ± 0.08 s^−^^1^) compared with the values determined for *Ca*LeuRS-Ser^919^ (*K*_m_: 1.87 ± 0.23 μM, *k_cat_*: 0.31 ± 0.05 s^−^^1^). These data indicated that *Ca*LeuRS-Ser^919^ has a stronger binding affinity for transcribed *Ca*tRNA^Leu^(UAA) during aminoacylation (Table 1). Based on the structure, we suggested that the presence of Ser in this helix may facilitate binding with the variable stem-loop element of *Ca*tRNA^Leu^(UAA). The catalytic efficiency of *Ca*LeuRS-Leu^919^ (213.06 s^−^^1 ^mM^−^^1^) is ∼30% higher than that of *Ca*LeuRS-Ser^919^ (165.78 s^−^^1 ^mM^−^^1^). This phenomenon is similar to that observed in the case of *Ca*SerRS, for which no differences were observed in the amino acid activation of *Ca*SerRS-Leu^197^ and *Ca*SerRS-Ser^197^, whereas *Ca*SerRS-Leu^197^ showed a slightly (27%) higher activity than *Ca*SerRS-Ser^197^(30).

**Table 1.** Aminoacylation kinetic parameters of *Ca*LeuRS-Ser^919^ and *Ca*LeuRS-Leu^919^ for the *Ca*tRNA^Leu^ transcript^a^

Enzyme	*K*_m_ (μM)	*k_cat_* (s^−1^)	*k_cat_*/*K*_m_ (s^−1 ^mM^−1^)	Relative *k_cat_*/*K*_m_
*Ca*LeuRS-Ser^919^	1.87 ± 0.23	0.31 ± 0.05	165.78	1.0
*Ca*LeuRS-Leu^919^	2.91 ± 0.37	0.62 ± 0.08	213.06	1.3

^a^The results are the average of three independent repeats with standard deviations indicated.

According to the decoding rule of *C. albicans*, *Ca*LeuRS is present in the cytoplasm mainly in the form of *Ca*LeuRS-Ser^919^. Thus, in the following study, we used *Ca*LeuRS-Ser^919^ as the wild-type *Ca*LeuRS.

### Yeast LeuRSs efficiently recognized bacterial, yeast and human tRNA^Leu^s

Species-specific charging of tRNA is common for some aaRSs systems. The aaRSs from higher organisms often have the capacity to charge tRNA from lower species, whereas aaRSs from lower organisms fail to aminoacylate tRNA from higher ones. It is unclear whether yeast LeuRS is able to recognize various tRNA^Leu^s from other species. In this study, we investigated the tRNA^Leu^ recognition capacity in detail using *Ca*LeuRS as a model system.

The *Ca*tRNA^Leu^ gene could not be over-expressed in *E. coli* and was obtained by T7 *in vitro* transcription. We also obtained transcribed and over-expressed *Ec*tRNA^Leu^ and hctRNA^Leu^ to reveal any potential role of base modification in recognition. Moreover, as transcribed *S. cerevisiae* tRNA^Leu^ without modification showed no Leu accepting activity (data not shown), commercial *S. cerevisiae* yeast total tRNA was used.

*Ca*LeuRS recognized all the available tRNAs. It aminoacylated transcribed or over-expressed *Ec*tRNA^Leu^ with similar *k_cat_* values (0.474 ± 0.026 and 0.555 ± 0.061 s^−^^1^, respectively), although the *K*_m_ for transcribed *Ec*tRNA^Leu^ (2.68 ± 0.39 μM) was nearly 4-fold greater than that for over-expressed *Ec*tRNA^Leu^ (0.74 ± 0.08 μM). Interestingly, *Ca*LeuRS efficiently charged both transcribed and over-expressed hctRNA^Leu^, which was from a higher organism. A similar recognition pattern as seen with the two *Ec*tRNA^Leu^s was also observed, with comparable *k_cat_* values but a smaller *K*_m_ for over-expressed hctRNA^Leu^, indicating base modification was important for tRNA recognition. Additionally, *Ca*LeuRS obviously charged yeast total tRNA with *K*_m_ and *k_cat_* values of 0.39 ± 0.05 μM and 0.174 ± 0.019 s^−^^1^, respectively, and with the greatest catalytic efficiency (1486.05 s^−^^1 ^mM^−^^1^) for over-expressed hctRNA^Leu^ among all the tested tRNAs (Table 2).

**Table 2.** Aminoacylation kinetic parameters of *Ca*LeuRS and *Sc*LeuRS for various tRNAs^a^

Enzyme	tRNA	*K*_m_ (μM)	*k_cat_* (s^−1^)	*k_cat_*/*K*_m_ (s^−1 ^mM^−1^)
*Ca*LeuRS	OE^b^-*Ec*tRNA^Leu^	0.74 ± 0.08	0.474 ± 0.026	640.54
	TS^c^-*Ec*tRNA^Leu^	2.68 ± 0.39	0.555 ± 0.061	207.09
	yeast total tRNA	0.39 ± 0.05	0.174 ± 0.019	446.15
	OE-hctRNA^Leu^	0.43 ± 0.05	0.639 ± 0.055	1486.05
	TS-hctRNA^Leu^	1.30 ± 0.23	0.719 ± 0.094	553.08
*Sc*LeuRS	OE-*Ec*tRNA^Leu^	2.19 ± 0.47	2.09 ± 0.16	954.34
	TS-*Ec*tRNA^Leu^	1.71 ± 0.22	0.134 ± 0.011	78.36
	yeast total tRNA	0.332 ± 0.037	0.188 ± 0.025	566.27
	OE-hctRNA^Leu^	0.111 ± 0.027	2.19 ± 0.22	19 729.73
	TS-hctRNA^Leu^	0.926 ± 0.170	0.887 ± 0.114	957.88

^a^The results are the average of three independent repeats with standard deviations indicated.

^b^OE, over-expressed.

^c^TS, transcribed.

Owing to recognition ability of *Ca*LeuRS for hctRNA^Leu^, we further explored the capacity of *Sc*LeuRS to aminoacylate bacterial and human tRNA^Leu^s as well as yeast tRNA. *Sc*LeuRS aminoacylated yeast total tRNA with *K*_m_ and *k_cat_* values of 0.332 ± 0.037 μM and 0.188 ± 0.025 s^−^^1^, respectively. However, its *k_cat_* values for over-expressed *Ec*tRNA^Leu^ or hctRNA^Leu^ increased >10-fold (2.09 ± 0.16 and 2.19 ± 0.22 s^−^^1^, respectively), although the *K*_m_ values differed from each other remarkably (2.19 ± 0.47 μM for over-expressed *Ec*tRNA^Leu^ and 0.111 ± 0.027 μM for over-expressed hctRNA^Leu^). These data demonstrated that over-expressed hctRNA^Leu^ was the best aminoacylation substrate for *Sc*LeuRS (catalytic efficiency 19 729.73 s^−^^1 ^mM^−^^1^) and furthermore suggested that base modification was important during recognition or catalysis. *Sc*LeuRS recognized transcribed *Ec*tRNA^Leu^ with a similar *K*_m_ (1.71 ± 0.22 μM) but a sharply decreased *k_cat_* (0.134 ± 0.011 s^−^^1^) compared with the values of over-expressed *Ec*tRNA^Leu^. It also recognized transcribed hctRNA^Leu^ with an increased *K*_m_ (0.926 ± 0.170 μM) and a decreased *k_cat_* (0.887 ± 0.114 s^−^^1^) compared with the values for over-expressed hctRNA^Leu^ (Table 2).

Overall, both *Ca*LeuRS and *Sc*LeuRS recognized bacterial, yeast and human tRNA^Leu^s. Interestingly, recognition of *Ca*tRNA^Leu^ by hcLeuRS was negligible (Supplementary Figure S1A). Futhermore, *Ec*LeuRS failed to acylate *Ca*tRNA^Leu^ (Supplementary Figure S1B). These results were unexpected because it is widely accepted that aaRSs from higher organisms are able to aminoacylate tRNAs from lower organisms.

### Amino acid activation capacity of CaLeuRS

**Figure 2.**
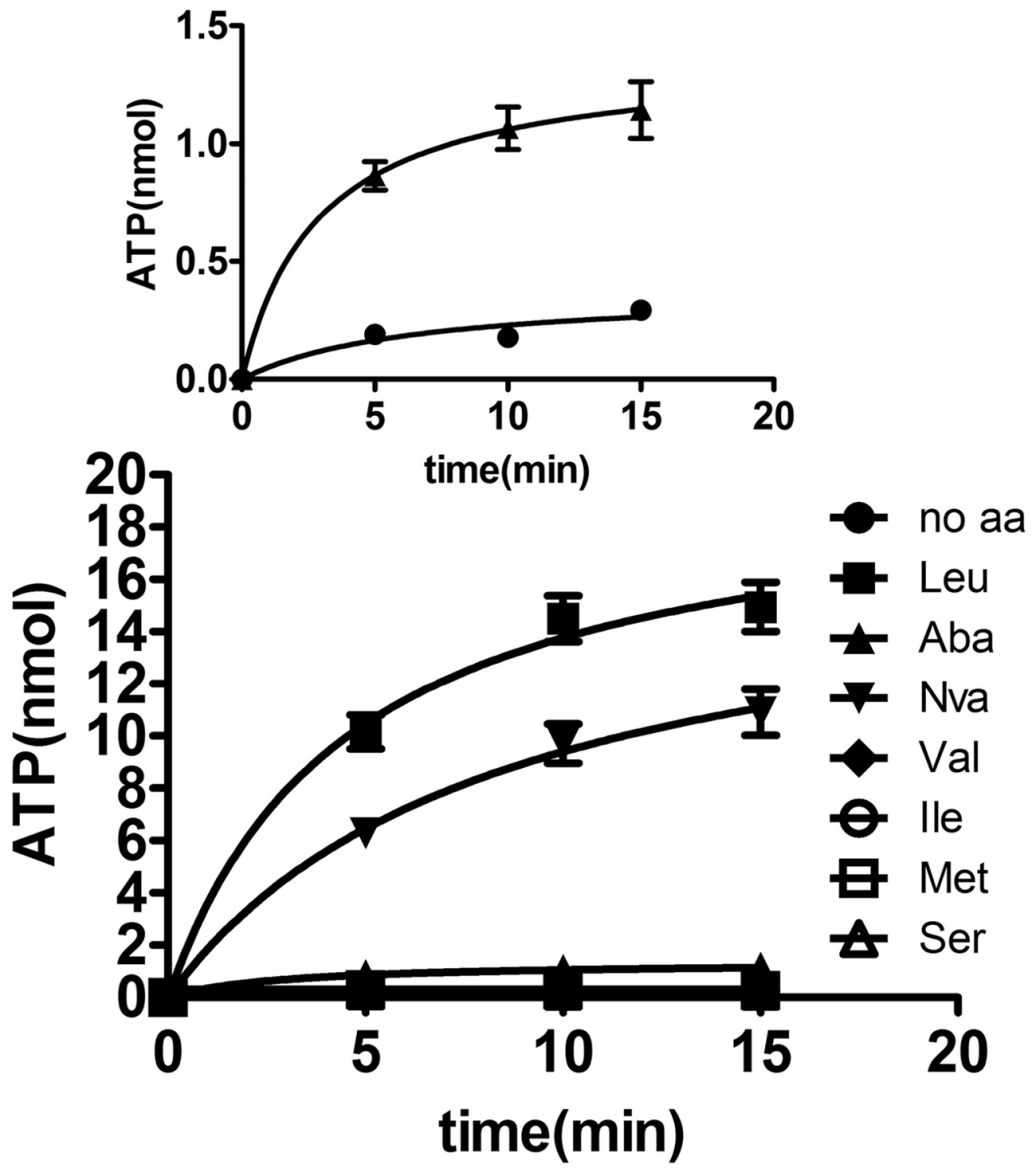
Amino acid activation properties of *Ca*LeuRS. Activation of Leu (black square), ABA (black up-pointing triangle), Nva (black down-pointing triangle), Val (black diamond), Ile (white circle), Met (white square) and Ser (white up-pointing triangle) by *Ca*LeuRS**.** Leu and non-cognate amino acids were used at final concentrations of 1 and 50 mM, respectively. A control reaction without any amino acids (black circle) was included. The insert compares the activation of ABA and the control.

Various LeuRSs have been shown to mis-activate a series of non-cognate amino acids. To investigate mis-activation of non-cognate amino acids by *Ca*LeuRS, we included ABA, Nva, Val, Ile, Met, Ser in the ATP-PPi exchange reaction. The data clearly showed that *Ca*LeuRS significantly mis-activated Nva; furthermore, ABA was also mis-activated to an obvious level compared with the control reaction conducted in the absence of amino acids. In contrast, mis-activation of Val, Ile, Met and Ser was comparable with that of the control reaction conducted in the absence of amino acids (Figure 2). To further define the quantitative discrimination capacity of the aminoacylation active site of *Ca*LeuRS, we measured the activation kinetics for cognate Leu and non-cognate Nva and ABA of *Ca*LeuRS. *Ca*LeuRS gave much higher *K*_m_ values for Nva (5487 ± 645 μM) and ABA (120387 ± 1698 μM); however, the *k_cat_* values were comparable with that for Leu, equating to discriminator factors for Nva and ABA of 220 and 3462, respectively (Table 3). These results indicated that Nva is a real challenge for *Ca*LeuRS and that removal of Nva-AMP and/or Nva-tRNA^Leu^ is required to maintain the translational quality control. However, the discrimination against ABA was below the proposed threshold of 1/3000, indicating that editing of ABA may not be necessary.

**Table 3.** Amino acid activation kinetics of *Ca*LeuRS for various amino acids^a^

Amino acid	*K*_m_ (μM)	*k_cat_* (s^−1^)	*k_cat_*/*K*_m_ (s^−1 ^mM^−1^)	Discrimination factor^b^
Leu	40.11 ± 3.84	93.05 ± 10.25	2319.87	1
Nva	5487 ± 645	57.77 ± 6.35	10.53	220
ABA	12 0387 ± 1698	80.28 ± 10.75	0.67	3462

^a^The results are the average of three independent repeats with standard deviations indicated.

^b^Discrimination factor corresponds to the loss of catalytic efficiency relative to Leu.

### *Ca*LeuRS exhibited little tRNA-dependent pre-transfer editing for Nva

The hydrolysis of Nva-AMP or Nva-tRNA^Leu^ may be separately or simultaneously catalyzed by *Ca*LeuRS. Editing leads to the net consumption of ATP (yielding AMP) due to repetitive cycles of synthesis-hydrolysis of the non-cognate products. This is the basis of the TLC-based AMP formation methodology, in which the editing capacity is measured by monitoring the quantity of AMP produced (9–11,36,37). In the presence of tRNA and non-cognate amino acid, the TLC assay measures the global editing activity, including the tRNA-independent and tRNA-dependent pre-transfer editing in addition to the post-transfer editing. In the absence of tRNA, but with non-cognate amino acid, AMP is produced only from tRNA-independent pre-transfer editing activity (10).

We initially assayed Nva-included AMP formation by *Ca*LeuRS with or without the *Ca*tRNA^Leu^ transcript. *Ca*LeuRS showed similar observed rate constants (*k*_obs_) with (0.28 ± 0.04 s^−^^1^) or without (0.25 ± 0.03 s^−^^1^) *Ca*tRNA^Leu^, indicating that *Ca*LeuRS possesses little tRNA-dependent editing capability (Table 4). To reveal any effect of the residue at position 919, we also determined the editing capacity of *Ca*LeuRS-Leu^919^ and obtained comparable *k*_obs_ values in the absence (0.22 ± 0.02 s^−^^1^) and presence (0.25 ± 0.05 s^−^^1^) of the *Ca*tRNA^Leu^ transcript. These data suggested that *Ca*LeuRS possesses negligible *Ca*tRNA^Leu^-dependent editing capability. It is also possible that modified bases of tRNA^Leu^ play a crucial role in Nva-editing. However, the lack of availability of over-expressed *Ca*tRNA^Leu^ impeded exploration of the potential role of modified bases in editing. Therefore, we performed AMP formation assays with Nva in the presence of transcribed or over-produced *Ec*tRNA^Leu^ in *E. coli*, which could be leucylated by *Ca*LeuRS. In accordance with our findings, the *k*_obs_ values with unmodified or modified *Ec*tRNA^Leu^ were 0.40 ± 0.06 or 0.51 ± 0.04 s^−^^1^, respectively. Similarly, transcribed or over-produced hctRNA^Leu^ in *E. coli*, both of which were effectively aminoacylated by *Ca*LeuRS, stimulated Nva-editing of *Ca*LeuRS with *k*_obs_ values of 0.33 ± 0.02 or 0.38 ± 0.05 s^−^^1^, respectively (Table 4). These data showed that the modified bases of tRNA^Leu^ had little effect on the tRNA-dependent editing of *Ca*LeuRS. Based on data from various transcripts or the tRNA^Leu^ with modified bases, we concluded that *Ca*LeuRS has little tRNA-dependent editing activity for Nva. Whether it was deficient in post-transfer editing would be explored later in the text. By comparing the *k*_obs_ values with or without tRNAs, we also observed that post-transfer editing, if it occurred, contributed little to the total editing, and that the observed *k*_obs_ with tRNAs was almost a reflection of the tRNA-independent pre-transfer editing.

**Table 4.** *k_obs_* values of *Ca*LeuRSs for editing Nva with various tRNAs

Enzyme	tRNA	*k*_obs_ (s^−1^)^a^
*Ca*LeuRS	No tRNA	0.25 ± 0.03
	*Ca*tRNA^Leu^	0.28 ± 0.04
	TS^b^-*Ec*tRNA^Leu^	0.40 ± 0.06
	OE^c^-*Ec*tRNA^Leu^	0.51 ± 0.04
	TS-hctRNA^Leu^	0.33 ± 0.02
	OE-hctRNA^Leu^	0.38 ± 0.05
*Ca*LeuRS-D422A	No tRNA	0.22 ± 0.06
	OE-hctRNA^Leu^	0.25 ± 0.04
*Ca*LeuRS-Leu^919^	No tRNA	0.22 ± 0.02
	*Ca*tRNA^Leu^	0.25 ± 0.05

^a^The results are the average of three independent repeats with standard deviations indicated.

^b^TS, transcribed.

^c^OE, over-expressed.

### *Ca*LeuRS exhibited obvious and efficient post-transfer editing to prevent synthesis of Nva-tRNA^Leu^

**Figure 3.**
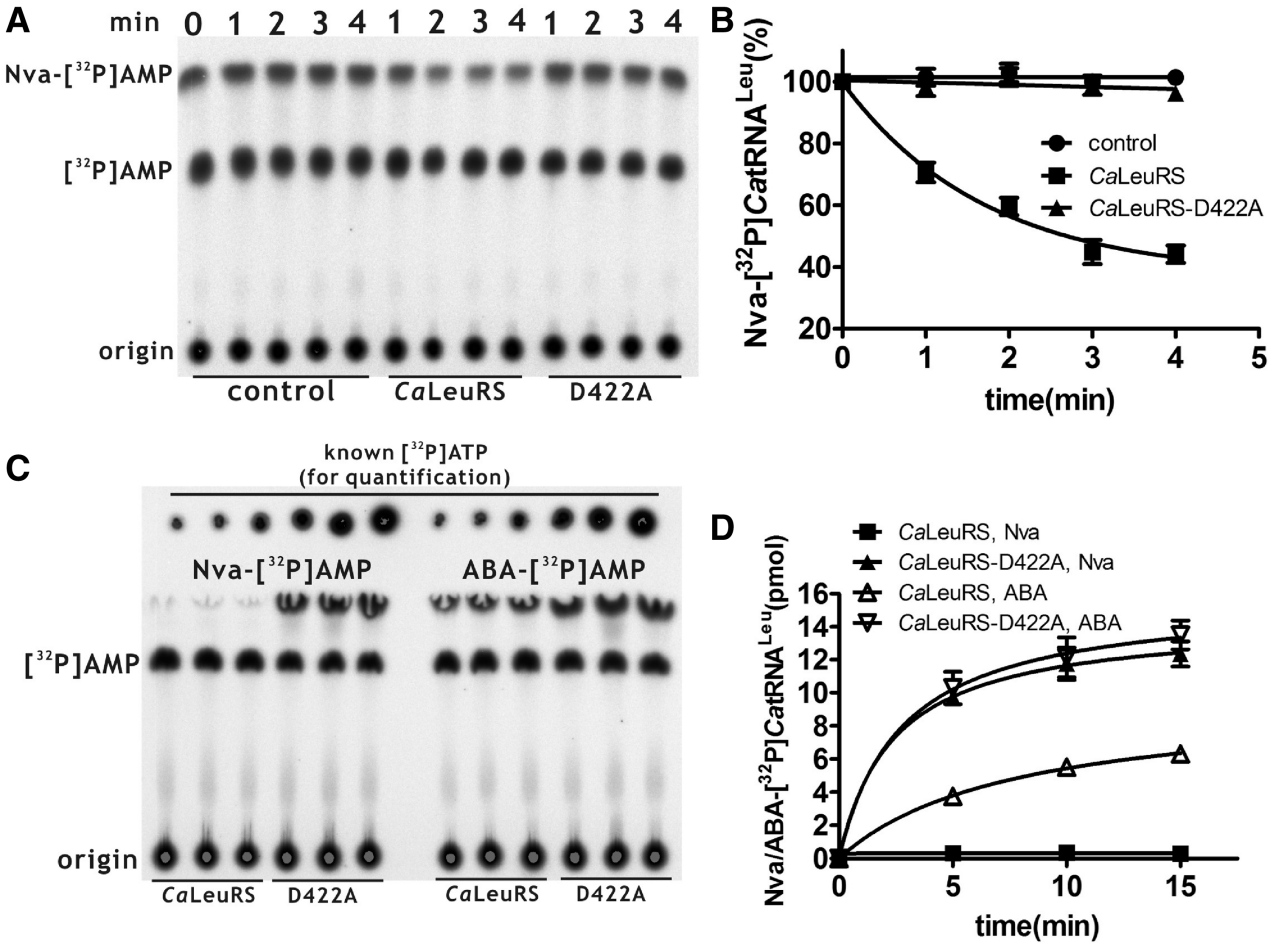
Post-transfer editing and mis-aminoacylation of *Ca*tRNA^Leu^ by *Ca*LeuRS and *Ca*LeuRS-D422A. (**A**) A representative graph showing hydrolysis of Nva-[^32^P]*Ca*tRNA^Leu^ by *Ca*LeuRS and *Ca*LeuRS-D422A. Nuclease S1-generated Nva-[^32^P]AMP (reflecting Nva-[^32^P]*Ca*tRNA^Leu^) and [^32^P]AMP (reflecting free [^32^P]*Ca*tRNA^Leu^) were separated by TLC. A control reaction represented the spontaneous hydrolysis of Nva-[^32^P]*Ca*tRNA^Leu^ without the addition of enzyme. (**B**) Analysis of post-transfer editing of Nva-[^32^P]*Ca*tRNA^Leu^ in (A). (**C**) A representative graph showing mis-charging of [^32^P]*Ca*tRNA^Leu^ with non-cognate Nva (left) or ABA (right). Free [^32^P]*Ca*tRNA^Leu^ and mis-charged [^32^P]*Ca*tRNA^Leu^ are represented by [^32^P]AMP and Nva-[^32^P]AMP or ABA-[^32^P]AMP, respectively. Known amounts of [α-^32^P]ATP were serially diluted and loaded onto the TLC plate after separation for quantification. (**D**) Quantitative analysis of Nva-[^32^P]*Ca*tRNA^Leu^ or ABA-[^32^P]*Ca*tRNA^Leu^ generated by *Ca*LeuRS (black square) and *Ca*LeuRS-D422A (black up-pointing triangle) or ABA-[^32^P]*Ca*tRNA^Leu^ by *Ca*LeuRS (white up-pointing triangle) and *Ca*LeuRS-D422A (white down-pointing triangle) in (C).

The absence of significant stimulation of editing of Nva by *Ca*LeuRS with various tRNA^Leu^s prompted us to investigate its post-transfer editing capability. Usually, the post-transfer editing ability of various LeuRSs is monitored by hydrolysis of Ile- or Met-tRNA^Leu^, which are easily obtained by mis-charging tRNA^Leu^ with commercially available radioactive Ile or Met using a LeuRS mutant without post-transfer editing capability. Because we focused on the Nva-editing properties of *Ca*LeuRS and Nva labeled with radioactive isotope was not commercially available, the 3′ end of *Ca*tRNA^Leu^ was first labeled with [α-^32^P]ATP by *E. coli* CCase, and then Nva-[^32^P]*Ca*tRNA^Leu^ was prepared by editing-deficient *Sc*LeuRS-D419A (13,18). Hydrolytic analysis clearly showed that *Ca*LeuRS edited Nva-[^32^P]*Ca*tRNA^Leu^ when compared with the control experiment conducted in the absence of the enzyme (Figure 3A and B). To confirm the post-transfer editing reaction catalyzed by *Ca*LeuRS, we mutated the conserved and post-transfer editing-essential Asp^422^ to generate *Ca*LeuRS-D422A. Asp^422^ corresponds to Asp^373^, Asp^419^, Asp^444^ and Asp^399^ of *Aa*LeuRS, *Sc*LeuRS, *Gl*LeuRS and hcLeuRS, respectively, which are crucial to post-transfer editing by these LeuRSs (9,10,18,19,27). Indeed, *Ca*LeuRS-D422A did not hydrolyze Nva-[^32^P]*Ca*tRNA^Leu^ and was deficient in post-transfer editing, indicating that this mutation inactivated the CP1 domain of *Ca*LeuRS (Figure 3A and B). Further mis-aminoacylation of [^32^P]*Ca*tRNA^Leu^ with Nva showed that a significant amount of Nva-[^32^P]*Ca*tRNA^Leu^ was formed by *Ca*LeuRS-D422A; however, a negligible amount of mis-charged *Ca*tRNA^Leu^ was formed by *Ca*LeuRS (Figure 3C and D). Overall, these data showed that *Ca*LeuRS harbored an obvious and efficient capability for post-transfer editing of Nva-[^32^P]*Ca*tRNA^Leu^, the loss of which caused accumulation of mis-charged tRNA^Leu^. No further accumulation of AMP after the addition of *Ca*tRNA^Leu^ in the AMP formation assay (Table 4) suggested that post-transfer editing of mis-charged *Ca*tRNA^Leu^ contributed little to the total editing. To further explore the absence of tRNA-dependent pre-transfer editing of Nva by *Ca*LeuRS, we tested the AMP formation of *Ca*LeuRS-D422A in the presence of Nva with or without over-produced hctRNA^Leu^. The data showed that over-produced hctRNA^Leu^ with modified bases did not stimulate further AMP production after abolishing post-transfer editing (k_obs_ 0.22 ± 0.06 versus 0.25 ± 0.04 s^−^^1^), confirming the lack of tRNA-dependent pre-transfer editing of Nva by *Ca*LeuRS (Table 4).

### *Ca*LeuRS inhibited synthesis of Nva-tRNA^Ser^ by non tRNA species-specific post-transfer editing

**Figure 4.**
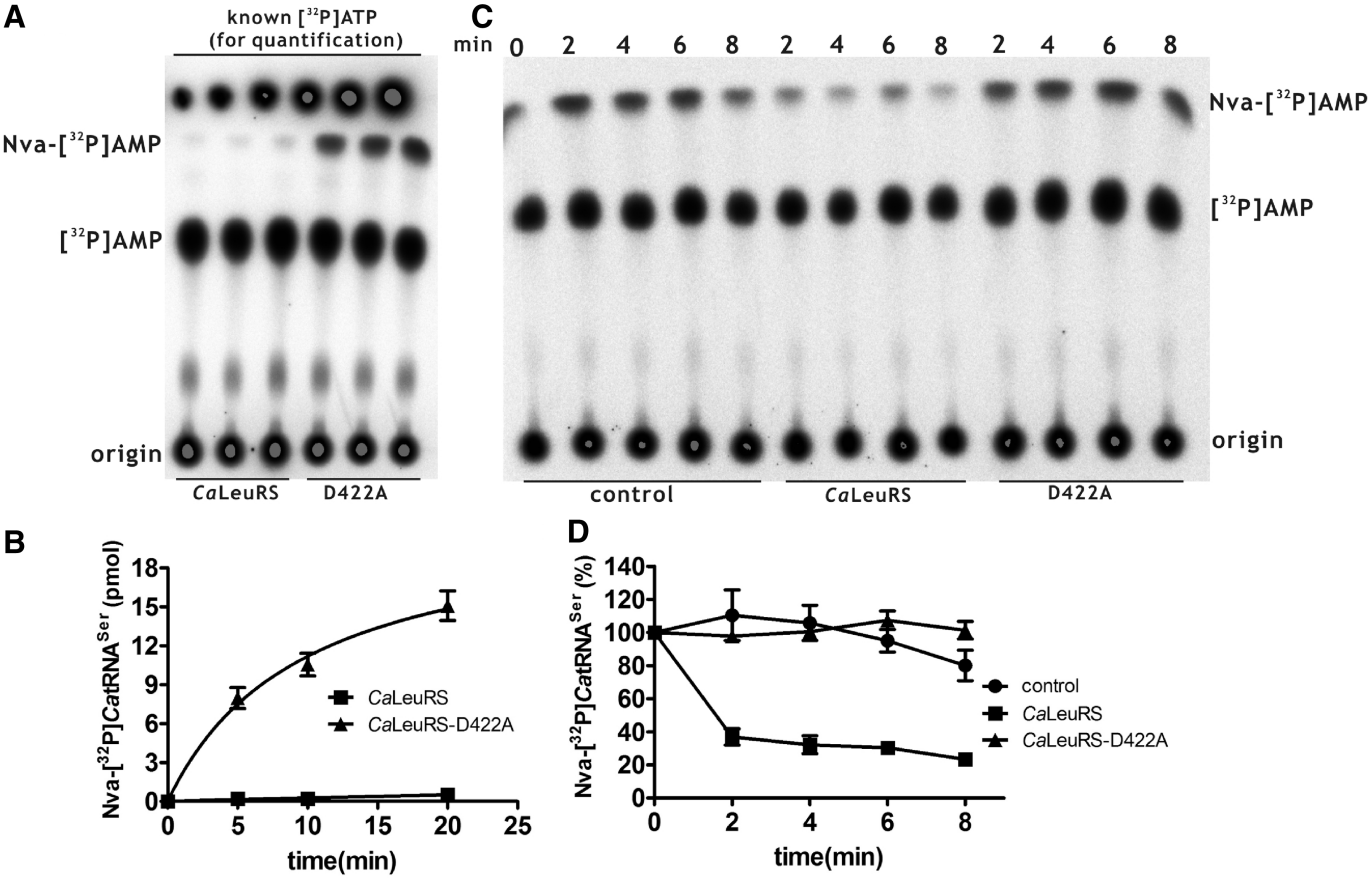
Mis-charging of [^32^P]*Ca*tRNA^Ser^ with Nva and post-transfer editing of Nva-[^32^P] *Ca*tRNA^Ser^ by *Ca*LeuRS and *Ca*LeuRS-D422A. (**A**) A representative graph showing mis-charging of [^32^P]*Ca*tRNA^Ser^ with non-cognate Nva. Free [^32^P]*Ca*tRNA^Ser^ and mis-charged [^32^P]*Ca*tRNA^Ser^ are represented by [^32^P]AMP and Nva-[^32^P]AMP after digestion of nuclease S1. Known amounts of [α-^32^P]ATP were serially diluted and loaded onto the TLC plate after separation for quantification. (**B**) Quantitative analysis of Nva-[^32^P] *Ca*tRNA^Ser^ generated by *Ca*LeuRS (black square) and *Ca*LeuRS-D422A (black up-pointing triangle) in (A). (**C**) A representative graph showing hydrolysis of Nva-[^32^P]*Ca*tRNA^Ser^ by *Ca*LeuRS and *Ca*LeuRS-D422A. A control reaction represented the spontaneous hydrolysis of Nva-[^32^P]*Ca*tRNA^Ser^ without the addition of enzyme. (**D**) Analysis of post-transfer editing of Nva-[^32^P]*Ca*tRNA^Ser^ by *Ca*LeuRS (black square) and *Ca*LeuRS-D422A (black up-pointing triangle) in (C).

The ability of *Ca*LeuRS to efficiently mis-activate non-cognate Nva and recognize non-cognate *Ca*tRNA^Ser^ raises the interesting question of how to prevent the formation of Nva-*Ca*tRNA^Ser^. To test for the presence of post-transfer editing activity that hydrolyzes potentially synthesized Nva-*Ca*tRNA^Ser^, mis-aminoacylation of [^32^P]*Ca*tRNA^Ser^ with Nva by *Ca*LeuRS was compared with that of the post-transfer editing-deficient *Ca*LeuRS-D422A. The data clearly showed that, mutation of Asp^422^ resulted in significant synthesis of Nva-[^32^P]*Ca*tRNA^Ser^ by the mutant, in contrast to wild-type enzyme, which generated negligible amounts of Nva-[^32^P]*Ca*tRNA^Ser^, indicating that *Ca*LeuRS used post-transfer editing to prevent Nva-*Ca*tRNA^Ser^ synthesis (Figure 4A and B). We then prepared Nva-*Ca*tRNA^Ser^ for use in hydrolysis assays to more directly monitor the post-transfer editing activity. Obvious hydrolysis of Nva-[^32^P]*Ca*tRNA^Ser^ was mediated by *Ca*LeuRS but not *Ca*LeuRS-D422A (Figure 4C and D). Above all, these data showed that the post-transfer editing by *Ca*LeuRS was not only *Ca*tRNA^Leu^ specific but also efficient for *Ca*tRNA^Ser^ to inhibit synthesis of both Nva-*Ca*tRNA^Leu^ and Nva-*Ca*tRNA^Ser^.

### *Ca*LeuRS possessed weak tRNA-dependent pre-transfer editing capacity for ABA

ABA was selected to test whether *Ca*LeuRS possessed any tRNA-dependent pre-transfer editing of other non-cognate amino acids because it was obviously activated by *Ca*LeuRS. *Ca*tRNA^Leu^ transcript, transcribed or over-produced *Ec*tRNA^Leu^ and hctRNA^Leu^ were used to trigger editing of ABA by *Ca*LeuRS (Table 5). The data showed that over-produced hctRNA^Leu^ obviously stimulated editing by increasing the *k*_obs_ 5-fold [(23.19 ± 3.62) × 10^−^^3 ^s^−^^1^] compared with that in the absence of tRNA [(4.69 ± 0.51) × 10^−^^3 ^s^−^^1^]. Over-expressed *Ec*tRNA^Leu^ led only to an ∼3-fold increase in *k*_obs_ [(14.58 ± 2.14) × 10^−^^3 ^s^−^^1^]. However, *Ca*tRNA^Leu^, *Ec*tRNA^Leu^ and hctRNA^Leu^ transcripts had little effect on the rate of ABA-editing (Table 5). These data implied that editing of ABA was tRNA modification-dependent. As over-produced hctRNA^Leu^ was the most efficient stimulator of ABA-editing, we measured AMP formation by the editing-deficient *Ca*LeuRS-D422A mutant in the presence of ABA with over-produced hctRNA^Leu^. Mutation of Asp^422^, which abolished post-transfer editing, apparently decreased the rate of AMP formation with a *k_obs_* of (12.16 ± 1.98) × 10^−^^3 ^s^−^^1^. Therefore, with over-produced hctRNA^Leu^, post-transfer editing of ABA by *Ca*LeuRS accounted for 47.6% of the total editing [(23.19 − 12.16)/23.19], whereas tRNA-independent and tRNA-dependent pre-transfer editing of ABA only accounted for 20.2% (4.69/23.19) and 32.2% [(12.16 − 4.69)/23.19], respectively, of the total editing of ABA by *Ca*LeuRS.

**Table 5.** *k_obs_* values of *Ca*LeuRSs for editing ABA with various tRNAs

Enzyme	tRNA	*k*_obs_ (×10^3^) (s^−1^)^a^
*Ca*LeuRS	No tRNA	4.69 ± 0.51
	*Ca*tRNA^Leu^	4.90 ± 0.37
	TS^b^-*Ec*tRNA^Leu^	4.78 ± 0.26
	OE^c^-*Ec*tRNA^Leu^	14.58 ± 2.14
	TS-hctRNA^Leu^	10.93 ± 0.85
	OE-hctRNA^Leu^	23.19 ± 3.62
*Ca*LeuRS-D422A	OE-hctRNA^Leu^	12.16 ± 1.98

^a^The results are the average of three independent repeats with standard deviations indicated.

^b^TS, transcribed.

^c^OE, over-expressed.

We further performed aminoacylation of [^32^P]*Ca*tRNA^Leu^ by *Ca*LeuRS and *Ca*LeuRS-D422A with saturating concentrations of ABA. The data showed that defective post-transfer editing resulted in the generation of significantly more ABA-[^32^P]*Ca*tRNA^Leu^; however, surprisingly, even *Ca*LeuRS yielded a significant amount of ABA-[^32^P]*Ca*tRNA^Leu^ (Figure 3C and D). These data implied that editing of ABA by *Ca*LeuRS was not sufficient to prevent the synthesis of ABA-[^32^P]*Ca*tRNA^Leu^ in the presence of saturating ABA concentrations. This paradox between ABA mis-aminoacylation and charging accuracy may be solved by fine discrimination against ABA at the aminoacylation active site (Table 3).

These results revealed that *Ca*LeuRS exhibits a weak level of tRNA-dependent pre-transfer editing activity for ABA. In addition, the total ABA-editing capacity is not sufficient to avoid the formation of mis-charged tRNA^Leu^, which is different from Nva-editing capacity.

### *Sc*LeuRS, like *Ca*LeuRS, also exhibited little tRNA-dependent pre-transfer editing capacity for Nva

The natural deficiency in tRNA-dependent pre-transfer editing of Nva by *Ca*LeuRS prompted us to investigate whether it is a common characteristic of other yeast LeuRS. Therefore, we assayed the Nva-included AMP formation catalyzed by *Sc*LeuRS in the absence or presence various tRNA^Leu^s. The data showed that over-produced hctRNA^Leu^ obviously stimulated editing (*k_obs_* of 0.64 ± 0.04 s^−^^1^) compared with that observed in the absence of tRNA (0.10 ± 0.02 s^−^^1^). However, transcribed *Ca*tRNA^Leu^, *Ec*tRNA^Leu^, hctRNA^Leu^ and over-expressed *Ec*tRNA^Leu^ failed to trigger further editing by *Sc*LeuRS (Table 6). The formation of AMP stimulated by tRNA^Leu^ should be derived from tRNA-dependent pre-transfer editing and/or post-transfer editing. To distinguish between these two pathways, the conserved Asp^419^ was mutated to generate *Sc*LeuRS-D419A, which has been shown to be defective in post-transfer editing and is homologous with Asp^422^ of *Ca*LeuRS (13,18). Assay of the Nva-included AMP formation by *Sc*LeuRS-D419A showed that the *k_obs_* with over-produced hctRNA^Leu^ was only slightly greater (0.100 ± 0.010 s^−^^1^) than that observed in the absence of tRNA (0.094 ± 0.001 s^−^^1^), indicating that inactivation of post-transfer editing totally abolished the triggering of AMP formation by tRNA, and that the increase in AMP production by over-produced hctRNA^Leu^ was due to post-transfer editing. Therefore, like *Ca*LeuRS, *Sc*LeuRS did not significantly catalyze tRNA-dependent pre-transfer editing for Nva.

**Table 6.** kobs values of ScLeuRSs for editing Nva with various tRNAs

Enzyme	tRNA	*k*_obs_ (s^−1^)^a^
*Sc*LeuRS	no tRNA	0.10 ± 0.02
	TS^b^-*Ec*tRNA^Leu^	0.10 ± 0.02
	OE^c^-*Ec*tRNA^Leu^	0.12 ± 0.03
	*Ca*tRNA^Leu^	0.10 ± 0.03
	TS-hctRNA^Leu^	0.10 ± 0.01
	OE-hctRNA^Leu^	0.64 ± 0.04
*Sc*LeuRS-D419A	no tRNA	0.094 ± 0.001
	OE-hctRNA^Leu^	0.100 ± 0.010

^a^The results are the average of three independent repeats with standard deviations indicated.

^b^TS, transcribed.

^c^OE, over-expressed.

### Eukaryotic, but not archaeal or bacterial LeuRSs, recognized CatRNA^Ser^

**Figure 5.**
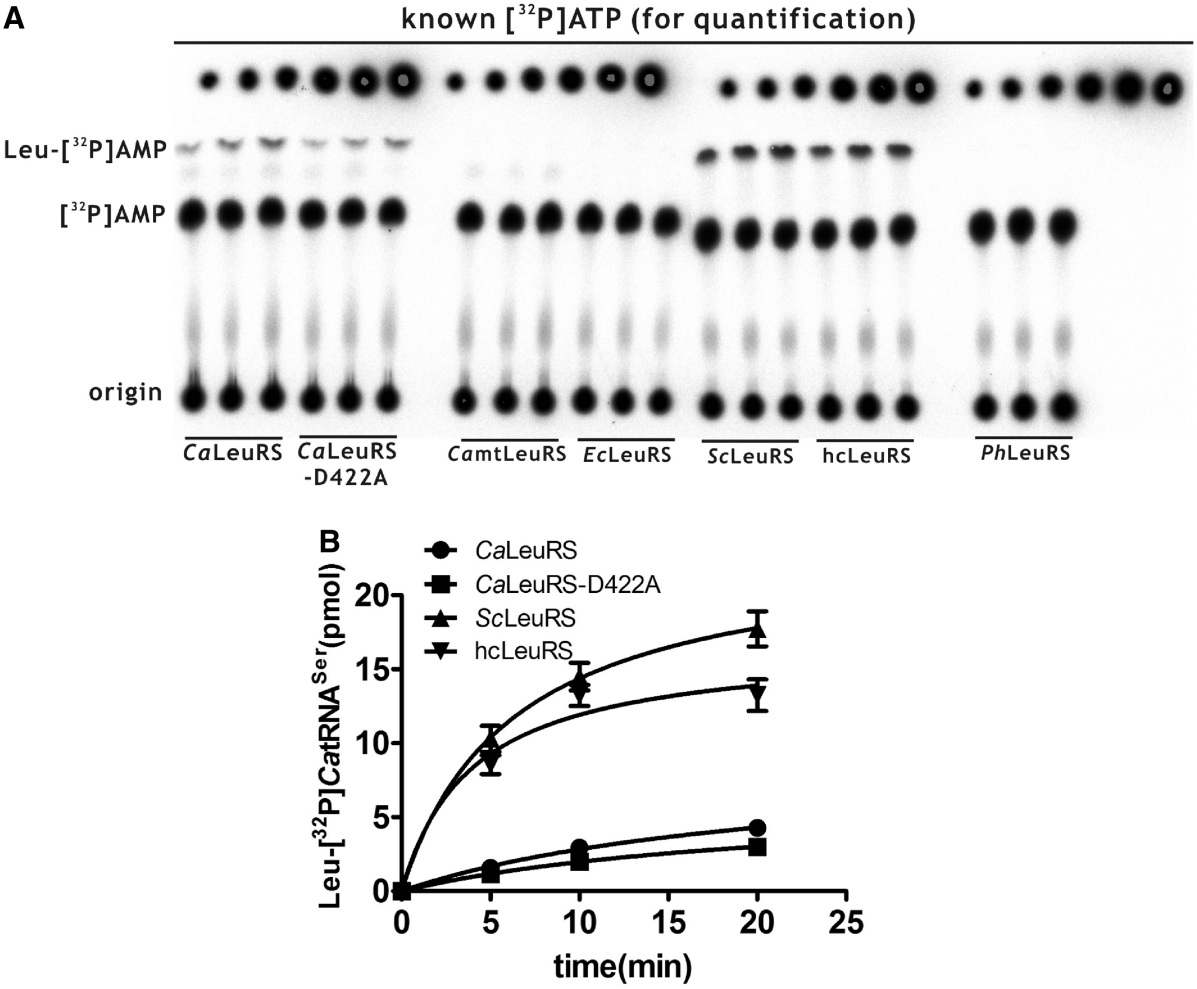
Recognition of *Ca*tRNA^Ser^ by representative bacterial, yeast, human and archaeal LeuRSs. (**A**) A representative graph showing aminoacylation of [^32^P]*Ca*tRNA^Leu^ with Leu by various LeuRSs. The generated Leu-[^32^P]*Ca*tRNA^Leu^ or free [^32^P]*Ca*tRNA^Leu^ was separated by TLC. Known amounts of [α-^32^P]ATP were serially diluted and loaded onto the TLC plate after separation for quantification. (**B**) Quantitative analysis of Leu-[^32^P]*Ca*tRNA^Ser^ generated by *Ca*LeuRS (black circle), *Ca*LeuRS-D422A (black square), *Sc*LeuRS (black up-pointing triangle) and hcLeuRS (black down-pointing triangle). No charged [^32^P]*Ca*tRNA^Ser^ was catalyzed by *Ca*mtLeuRS, *Ec*LeuRS and *Ph*LeuRS.

It is interesting that LeuRSs from some *Candida* species recognize a uniquely evolved tRNA^Ser^ to introduce ambiguity at CUG codons (28). Unfortunately, no elements of *Ca*LeuRS have been identified to account for the interaction with anti-codon stem and/or loop of *Ca*tRNA^Ser^. To test whether other eukaryotic, archaeal, bacterial or mitochondrial LeuRSs could potentially recognize *Ca*tRNA^Ser^, we performed aminoacylation of [^32^P]*Ca*tRNA^Ser^ with Leu by *Ca*mtLeuRS, *Ec*LeuRS, *Sc*LeuRS, hcLeuRS and *Ph*LeuRS. The data showed that only eukaryotic LeuRSs (including *Ca*LeuRS, *Sc*LeuRS and hcLeuRS) could aminoacylate *Ca*tRNA^Ser^ with Leu; however, other LeuRSs, including bacterial *Ec*LeuRS, mitochondrial *Ca*mtLeuRS and archaeal *Ph*LeuRS, failed to charge it (Figure 5). Strikingly, under the same conditions, *Sc*LeuRS and hcLeuRS mediated more efficient aminoacylation of *Ca*tRNA^Ser^.

## DISCUSSION

### Insertion of Ser or Leu at CUG codons might not be incidental

In *C. albicans* and most other CUG clade species, a mutant tRNA^Ser^(CAG) has evolved to decode the Leu CUG codon both as Ser and Leu (28,29). This peculiarity is derived from its combined tRNA^Leu^ and tRNA^Ser^ identity elements (38). This tRNA is mainly aminoacylated by SerRS and charged by LeuRS to a small extent (29). Both biochemical and structural data have revealed that ambiguity at the single CUG codon of SerRS induces local structural rearrangement, leading to a slightly increased activity (27%) of *Ca*SerRS-Leu^197^ compared with the wild-type *Ca*SerRS-Ser^197^ (30). Furthermore, genetic studies showed that increased Leu incorporation across all the CUG codons of *C. albicans* had no visible effect on the growth phenotype but had an impressive impact on cell morphology (39). Therefore, it was proposed that CUG decoding ambiguity has a potential regulatory role in protein structure and/or function (30). *Ca*LeuRS is another crucial player in this genetic code alteration and also contains only one CUG codon at position 919. This site is located at the C-terminal domain of LeuRS, which has been shown to be responsible for binding the variable loop of tRNA^Leu^ and involved in the aminoacylation activity; however, this domain is not strictly conserved among archaeal/eukaryotic LeuRSs (Figure 1A and B). Here, we revealed that both *Ca*LeuRS-Leu^919^ and *Ca*LeuRS-Ser^919^ catalyzed Leu activation and aminoacylation, but the former was more active (30%) than the latter, indicating that the conformation of the 919-containing α29 helix might be finely controlled by the introduction of either Ser or Leu. This phenomenon was also observed in another crucial player in the CUG decoding alteration pathway, *Ca*SerRS (30). We suggested that insertion of either Ser or Leu at the CUG codon was not random and incidental. The relative amounts of *Ca*LeuRS-Ser^919^/*Ca*LeuRS-Leu^919^ should be strictly regulated by an unidentified but precise molecular mechanism *in vivo*. Whether the fine balance of *Ca*LeuRS-Ser^919^/*Ca*LeuRS-Leu^919^ is critical for decoding other Leu codons and correlates with the ratio of *Ca*SerRS-Ser^197^/*Ca*SerRS-Leu^197^ requires further investigation.

### Yeast LeuRS exhibited a relaxed tRNA recognition capacity

In tRNA aminoacylation, species-specific charging, where a tRNA from one taxonomic domain is not aminoacylated by an aaRS from another, is widespread. This may be as a result of the co-evolution of synthetase/tRNA pairs by the addition of species-specific elements. For instance, human tyrosyl-tRNA synthetase does not recognize bacterial tRNA^Tyr^, and *E. coli* tyrosyl-tRNA synthetase is unable to charge eukaryotic tRNA^Tyr^ (40), and there is no cross-recognition of *E. coli* and human tRNA^Gly^ by the respective glycyl-tRNA synthetases (41). Similarly, *E. coli* isoleucyl-tRNA synthetase is unable to charge eukaryotic tRNA^Ile^ (42). Yeast ArgRS charges *E. coli* tRNA^Arg^; however, *E. coli* ArgRS acylates only its cognate *E. coli* tRNA (43). Human cysteinyl-tRNA synthetase charges bacterial tRNA^Cys^, but *E. coli* cysteinyl-tRNA synthetase is non-functional in aminoacylating human tRNA^Tyr^ (44). Here, we showed that both hcLeuRS and *Ec*LeuRS failed to aminoacylate *Ca*tRNA^Leu^; however, both *Ca*LeuRS and *Sc*LeuRS readily aminoacylated bacterial, yeast and even human tRNA^Leu^s. These results showed that yeast LeuRSs exhibit a more relaxed recognition specificity compared with other LeuRSs. Indeed, *Ca*tRNA^Ser^ itself harbors only tRNA^Leu^ recognition elements in the anti-codon loop with other parts being crucial for SerRS recognition. In addition, G33 is also unfavorable for LeuRS; even in this adverse state, *Ca*LeuRS aminoacylates it *in vivo* (29). Comparison between transcribed and over-expressed tRNA^Leu^s showed that base modification of tRNA^Leu^ plays an important role in both binding and catalysis.

### *Ca*LeuRS was deficient in tRNA-dependent pre-transfer editing but exhibited efficient post-transfer editing for Nva

Nva is inherently mis-activated by various LeuRSs to a significant level that requires editing for translational accuracy (9,18–20). With an elevated ratio of Nva to Leu, Nva can escape the safeguarding of *Ec*LeuRS and replace Leu in proteins rich in Leu codons, indicating that Nva-tRNA^Leu^ can escape further checking by the ribosome and pose a direct threat to the accuracy of newly synthesized proteins (24). From the viewpoint of editing, some LeuRSs with degenerated (e.g. hmtLeuRS) or deleted CP1 (e.g. *Mm*LeuRS) domains are exceptional examples, which use alternative pathways (efficient discrimination at the active site) for translational quality control (hmtLeuRS) (26) or do not edit mis-aminoacylation product to produce proteome ambiguity (*Mm*LeuRS) (20). However, all LeuRSs with functional CP1 domains studied so far display tRNA-independent, tRNA-dependent pre-transfer editing and post-transfer editing for Nva. Through inactivation of CP1 or utilization of LeuRS inhibitors, post-transfer editing has been successfully isolated (9,10,18,19,27). Similarly, by mutating a crucial Tyr residue to Asp in *Ec*LeuRS and *Aa*LeuRS, both tRNA-dependent pre-transfer and post-transfer editing are inactivated (10,33). Interestingly, this study identified that *Ca*LeuRS itself is naturally defective in tRNA-dependent pre-transfer editing for Nva. With *Ca*tRNA^Leu^, no tRNA-dependent pre-transfer editing was identified. In contrast, weak tRNA-dependent pre-transfer editing for ABA in the presence of specific tRNA^Leu^ was observed, despite the indication that ABA-editing might not be necessary *in vivo* based on fine discrimination at the active site. Similarly, *Sc*LeuRS did not mediate tRNA-dependent pre-transfer editing. These results indicate that the capacity for tRNA-dependent pre-transfer editing for Nva has been lost by *Ca*LeuRS (also *Sc*LeuRS), and that ABA is also rarely induced. The reason for this deficiency in tRNA-dependent pre-transfer editing and the pathway by which this deficiency was introduced remains to be elucidated.

Post-transfer editing contributed little or negligibly to the total Nva-editing since addition of any tRNAs in the AMP formation assays did not significantly induce additional AMP. Thus, the produced AMP was mainly derived from tRNA-independent pre-transfer editing. However, the energy-saving post-transfer editing pathway critically controls the accuracy of aminoacylation. Mutation at the conserved Asp^422^ of *Ca*LeuRS led to a LeuRS with abolished post-transfer editing capacity; consequently, Nva-tRNA^Leu^ was synthesized. Similarly, *Sc*LeuRS did not synthesize Ile-tRNA^Leu^; however, *Sc*LeuRS-D419A readily generated significant amounts of Ile-tRNA^Leu^ (13,18). Using these unique *Ca*LeuRS and *Sc*LeuRS models devoid of tRNA-dependent pre-transfer editing capacity, we concluded that the post-transfer editing pathway is the most economic but efficient editing mechanism for LeuRS. Consistent with other LeuRS models and even other aaRS systems, once post-transfer editing is impaired, the mis-charged tRNA is unavoidably accumulated (9–11,18,19,27,45,46).

Our results also revealed that post-transfer editing by *Ca*LeuRS is not tRNA-species specific, as Nva-*Ca*tRNA^Ser^ was also a substrate. Indeed, based on the poor discrimination against Nva in the active site, Nva-tRNA^Ser^ is possibly synthesized but should be removed. Otherwise, the CUG codon might be decoded as Ser, Leu and Nva *in vivo*. It has been proposed that the acceptor end of the tRNA switches from a hairpin conformation to a helical conformation for editing by class I aaRSs, whereas the reverse change in conformation occurs at the acceptor end of the tRNA for editing by class II aaRSs (47). Notably, *Ca*tRNA^Ser^ corresponds to a class II SerRS; however, results here showed that *Ca*tRNA^Ser^ could switch from a hairpin to a helical conformation for editing by a class I LeuRS.

### Eukaryotic LeuRSs recognized CatRNA^Ser^

In addition, we revealed that other eukaryotic LeuRSs could efficiently aminoacylate *Ca*tRNA^Ser^, implying that the introduction or evolution of this type of tRNA in other eukaryotic systems would reprogram or discombobulate the genetic code, leading to proteome chaos. In other words, a specific eukaryotic genetic code could be artificially reprogramed by expression of this tRNA^Ser^. Indeed, *Ca*tRNA^Ser^ has been shown to be efficiently produced, processed and aminoacylated in *S. cerevisiae*, with its expression triggering a stress response, blocking mating and re-defining the gene expression model of *S. cerevisiae* (48). Notably, archaeal LeuRS is in the same group with eukaryotic LeuRS according to primary or higher structure (14) and only differs at the C-terminal tRNA binding domain, indicating that this domain in eukaryotic LeuRSs is a key element for recognition of *Ca*tRNA^Ser^. This observation is consistent with the structural and biochemical results showing that the C-terminal domain of archaeal LeuRS specifically contacts the variable loop but not the anti-codon loop of archaeal tRNA^Leu^ (16,49). However, the anti-codon loop, which is a key recognition element in both *Ca*tRNA^Ser^ (28,29,38) and yeast tRNA^Leu^ (49), is likely to be bound by the C-terminal domain of eukaryotic LeuRS. This proposal requires confirmation from eukaryotic LeuRS-tRNA^Leu^/tRNA^Ser^ structures.

### Concluding remarks

Translational machinery of human pathogen *C. albicans* is of particular interest because its CUG codon in the genome is decoded as both Ser and Leu by a unique *Ca*tRNA^Ser^, leading to proteome ambiguity (28,29). One of the most crucial components in this decoding process is *Ca*LeuRS, which catalyzes two successive steps, aminoacylation and editing reactions, which together are essential for ensuring high specificity of tRNA charging. In aminoacylation, we showed that Leu isoform was more active than Ser isoform of *Ca*LeuRS in charging *Ca*tRNA^Leu^, implying the existence of an *in vivo* mechanism regulated by balance of *Ca*LeuRS-Leu^919^ and *Ca*LeuRS-Ser^919^. In addition, as a yeast LeuRS model, *Ca*LeuRS recognized tRNA^Leu^s from bacteria, yeast and higher eukaryote. In editing, *Ca*LeuRS efficiently mis-activated non-cognate Nva. One of the most interesting findings was that *Ca*LeuRS lacked tRNA-dependent pre-transfer editing for Nva, which has been well investigated for bacterial and human LeuRSs (10,19). Instead, *Ca*LeuRS prevented insertion of Nva into proteome mainly via post-transfer editing no matter whether Nva has been loaded onto either *Ca*tRNA^Leu^ or *Ca*tRNA^Ser^. Collectively, we further improved our understanding of mechanism and significance of genetic code ambiguity in *C. albicans* and revealed interesting properties of both aminoacylation and editing reactions by *Ca*LeuRS. Furthermore, the capacity of eukaryotic LeuRSs at aminoacylating *Ca*tRNA^Ser^ suggests the possibility of reconstructing proteome of other eukaryotes by simply introducing this unique tRNA^Ser^.

## SUPPLEMENTARY DATA

Supplementary Data are available at NAR Online.

## FUNDING

Natural Science Foundation of China [31270852, 31000355]; National Key Basic Research Foundation of China [2012CB911000]. Funding for open access charge: [31270852, 2012CB911000].

*Conflict of interest statement*. None declared.

## Supplementary Material

Supplementary Data
