# Supplementary material for: Aminoacylation and translational quality control strategy employed by leucyl-tRNA synthetase from a human pathogen with genetic code ambiguity
Source: Nucleic Acids Res. 2013 Aug 22;41(21):9825–38. doi: 10.1093/nar/gkt741 (PMC3834818; doi:10.1093/nar/gkt741)
Supplement: Supplementary Data [file supp_41_21_9825__index.html]

Aminoacylation and translational quality control strategy employed by leucyl-tRNA synthetase from a human pathogen with genetic code ambiguity — Aminoacylation and translational quality control strategy employed by leucyl-tRNA synthetase from a human pathogen with genetic code ambiguity — Supplementary Data 

# Aminoacylation and translational quality control strategy employed by leucyl-tRNA synthetase from a human pathogen with genetic code ambiguity

## Supplementary Data

files

**Files in this Data Supplement:**

- Supplementary Data - docx file
