# Supplementary material for: Aminoacylation and translational quality control strategy employed by leucyl-tRNA synthetase from a human pathogen with genetic code ambiguity
Source: Nucleic Acids Res. 2013 Aug 22;41(21):9825–38. doi: 10.1093/nar/gkt741 (PMC3834818; doi:10.1093/nar/gkt741)
Supplement: Supplementary Data [file supp_gkt741_nar-01703-r-2013-File003.docx]

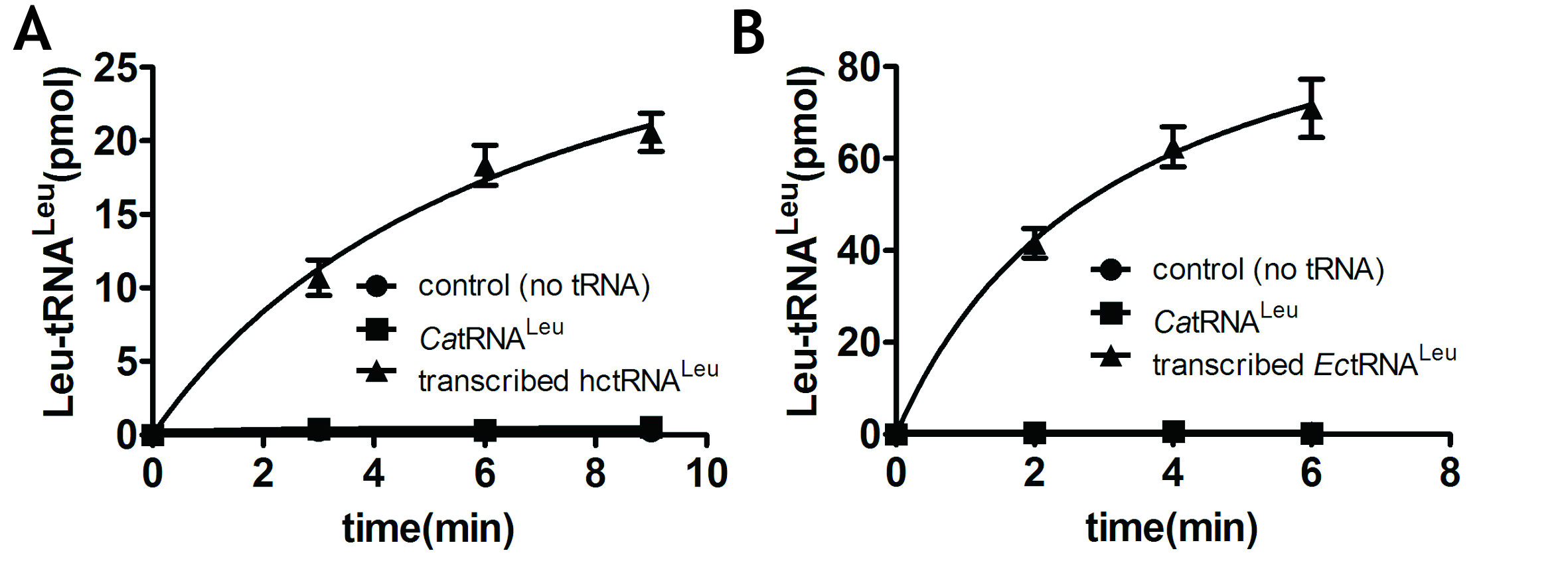


**Supplementary Figure 1**

**Supplementary Figure 1: Recognition of *Ca*tRNA^Leu^ by hcLeuRS and *Ec*LeuRS.**

(A) Aminoacylation of *Ca*tRNA^Leu^ (■) and transcrived hctRNA^Leu^ (▲) by hcLeuRS. (B) Aminoacylation of *Ca*tRNA^Leu^ (■) and transcrived *Ec*tRNA^Leu^ (▲) by *Ec*LeuRS. Controls were included without addition of tRNA.
